# Seroepidemiology of Maedi-Visna in Intensively Reared Dairy Sheep: A Two-Year Prospective Study

**DOI:** 10.3390/ani13142273

**Published:** 2023-07-12

**Authors:** Aphrodite I. Kalogianni, Ilias Bouzalas, Ioannis Bossis, Athanasios I. Gelasakis

**Affiliations:** 1 Department of Animal Science, School of Animal Biosciences, Agricultural University of Athens, 11855 Athens, Greece; gelasakis@aua.gr; 2 Veterinary Research Institute, Hellenic Agricultural Organization—DEMETER, Campus of Thermi, 57001 Thessaloniki, Greece; bouzalas@elgo.gr; 3 Department of Agricultural Sciences, School of Agriculture, Forestry and Natural Resources, University Campus, Aristotle University of Thessaloniki, 54124 Thessaloniki, Greece; bossisi@agro.auth.gr

**Keywords:** maedi-visna, prospective seroepidemiological study, seroconversion, seroreversion, intermittent presence of antibodies

## Abstract

**Simple Summary:**

Maedi-visna is a globally distributed, viral, and incurable chronic disease of small ruminants, challenging the health and welfare of sheep and jeopardizing the sustainability of farms. Currently, the scarcity of representative, updated, and epidemiological data hinders the implementation of successful eradication programs. The objective of our study is to prospectively study the seroepidemiology of maedi-visna in intensively reared dairy sheep. For this reason, a total of 407 purebred Chios and Lacaune ewes from four farms were studied for two successive years, and blood samples were semiannually collected and tested using the ELISA test. Prevalence and incidence rates were found to have increased in all the studied farms, highlighting the importance of horizontal transmission of the virus within the farms. Various serological patterns were observed in the studied animals, namely, constantly seronegative, constantly seropositive, seroconverted, seroreverted, or animals with an intermittent presence of antibodies. The last two categories indicate a special immune response of infected animals which needs to be further investigated and considered for the diagnosis of MV and when designing eradication programs.

**Abstract:**

The objective of this study is to prospectively evaluate the seroepidemiology of maedi-visna (MV) infections in intensively reared dairy sheep. A total of 407 purebred Chios and Lacaune ewes from four farms were surveyed for two consecutive years and were serologically tested semiannually with an indirect ELISA at pre-mating and pre-lambing. The farms’ structure and management practices were similar and animal traits (age, breed, and production stage) were recorded. Based on the serological status, morbidity frequency measures were estimated, and ewes were categorized as constantly seronegative, constantly seropositive, seroconverted, seroreverted, or as animals with an intermittent presence of antibodies. During the study, period seroprevalence, incidence rate, and cumulative incidence were 84.8%, 33.6 new cases per 100 sheep-semesters, and 64.2%. Point-seroprevalence ranged from 48.5% to 96.0% among the studied farms and sampling occasions, and they increased by age. Increased morbidity frequency measures indicate the significance of horizontal transmission in intensive dairy sheep farms. A remarkable percentage of infected animals seroreverted (8.1%) or presented an intermittent presence of antibodies (10.3%) during the study, confirming the risk of misdiagnosis in cross-sectional studies and in the currently implemented testing and elimination programs. The serological patterns observed in our study need to be considered when studying MV epidemiology and for the designing of efficient MV elimination programs.

## 1. Introduction

Maedi-visna (MV) is a chronic incurable disease of sheep caused by a small ruminant lentivirus, namely the maedi-visna virus (MVV) of the family *Retroviridae* [1]. The maedi-visna virus causes a lifelong infection in sheep with a long incubation period leading to latent infection without clinical symptoms even for years [2]. Clinical manifestations associated with MVV infections include pneumonia, mastitis, arthritis, and encephalitis [3], while progressive body weight loss and, in some cases, death of the infected sheep may occur [4]. As a result, MV challenges the health and welfare of animals and jeopardizes the sustainability of sheep farms, leading to substantial economic losses due to increased culling and replacement rate as well as decreased milk production and lamb growth [5,6,7,8].

MVV has a global spread, and preventive measures against its transmission between and within the farms were proved ineffective at various degrees. Among others, this is associated with an evident lack of updated epidemiological data on MV, which renders the development of efficient control strategies a challenging endeavor. Formal data regarding MV prevalence in European countries are insufficient and derive mainly from limited voluntary epidemiological studies contacted in specific regions and breeds, including animals reared under dissimilar farming systems rather than from systematically applied national surveillance programs. However, these studies may not be representative of the current situation; the inclusion of MV in the list of notifiable terrestrial and aquatic animal diseases by the World Organisation for Animal Health (WOAH), and the subsequent limitations in the trading of breeding stocks, discourages sheep breeders from voluntarily investigating the presence of the disease in their farm.

Currently, investigation of MV prevalence is based on cross-sectional epidemiological studies. According to these studies, seroprevalence values vary among countries, farming systems, age classes, and breeds of the studied animals and highly depend on the applied laboratory tests [5,9,10,11,12,13,14,15,16,17,18]. However, results from this type of studies are not always reliable due to the underdiagnosis of infected animals, as some of them may remain constantly seronegative from months to years after the infection, demonstrate fluctuating antibody titers, or even serorevert [19]. Underdiagnosis of infected animals leads to their misclassification as being uninfected, undermining the successful implementation of control programs and hampering the eradication of MV.

The scarcity of epidemiological data on MV does not allow the designation of evidence-based control programs, which should be precisely adapted per region and farming system, according to the morbidity frequency measures and the potential risk factors (genetic predisposition of breeds, management, and productive orientation). In Greece, although dairy sheep farming is a major sector of livestock production and there is evidence of extensive MV virus spread, the lack of updated epidemiological data does not allow the proposal and implementation of targeted national control programs.

The objectives of this study are to prospectively study the seroepidemiology of MV infections, to calculate morbidity frequency measures, and to determine serological patterns in MV naturally infected ewes in intensive dairy sheep farms in Greece.

## 2. Materials and Methods

### 2.1. Animal Population and Study Design

**Figure 1:**
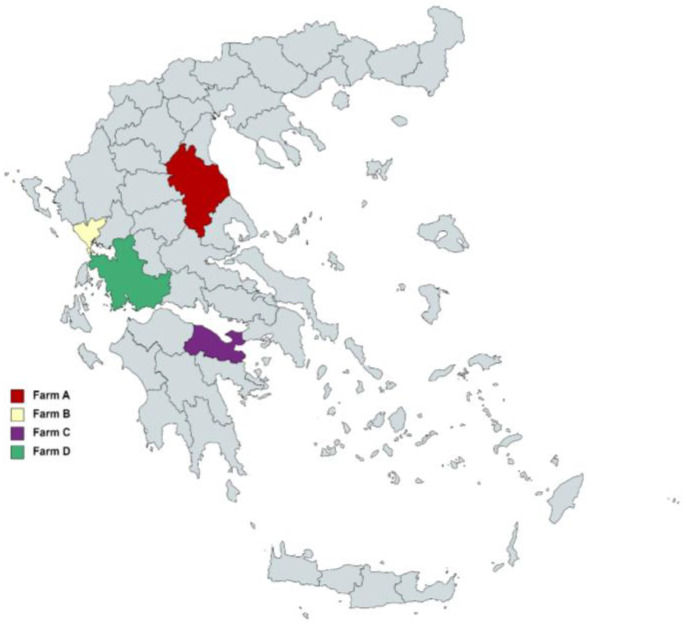
Geographical distribution of the studied farms.

A total of ten intensive, zero-grazing dairy sheep farms were initially surveyed during on-site visits and interviews with the farmers, using a structured questionnaire to collect data regarding farms’ characteristics and management practices. Among them, four intensive dairy sheep farms with purebred Chios (farm A, B, and C) and Lacaune sheep (farm A and farm D)—located at different counties in Greece (Figure 1)—were selected and enrolled in the study, on the basis of (i) being representative of the intensive system [20], (ii) applying similar management schemes (Table 1), (iii) being recently found to be seropositive of MV, and (iv) fulfilling the terms of participation and collaboration during the whole duration of the study. From these farms, a total of 660 randomly selected ewes (6 months to 7 years old) were included in the study which initiated 2 to 4 weeks pre-mating. The selected animals were separately penned and prospectively studied for two consecutive years.

### 2.2. Blood Sampling and Serological Analysis

Samplings were performed semiannually for two consecutive years, in specific physiological stages of the production life of sheep, namely 3–4 weeks pre-mating and 2–4 weeks pre-lambing. The protocol of this study was approved by the Animal Research Ethics Committee of the Agricultural University of Athens and was in accordance with the national animal welfare regulations. Blood samples were collected in clot activator tubes by the same trained veterinarian and transferred under 4 °C in the lab where they were centrifuged at 3000× *g* for 10 min; the serum was separated and used for the detection of anti-MVV antibodies utilizing an indirect commercial ELISA test (ELISA, CAEV/MVV Total Ab Test, IDEXX). Based on the literature, the analytical sensitivity and specificity values of the test ELISA are 95.5% and 97.2%, respectively, when compared to a recombinant GAG (group-specific antigens)–GST (glutathione S-transferase) fusion protein expressed in *E. coli* ELISA [21]. Relative optical density (OD) values of ELISA were calculated using the formula:

Relative OD value = 100 × (OD_sample_ − OD_negative control_)/(OD_positive control_ − OD_negative control_) (1)

According to the manufacturer’s instruction, samples were considered positive when relative OD values were greater than 60 and suspect when relative OD values were between 50 and 60. In our study, suspect results were considered as seropositive, and the cut-off value was set at 50. The ear tag, breed, and age were recorded for each individual ewe.

### 2.3. Epidemiological and Statistical Analysis

From the initially enrolled animals, only ones with at least four consecutive samplings were retained, and their data were used for the statistical analyses. This resulted in a total of 407 animals (234 Chios: 25, 143, and 66 from farms A, B, and C, respectively; 173 Lacaune: 32 and 141 from farms A and D, respectively) with a full set of measurements. These animals were categorized according to their serological pattern as constantly seropositive (exclusively seropositive results during the study), constantly seronegative (exclusively seronegative results during the study), seroconverted (seronegative animals at the beginning of the study which converted to seropositive during the study), seroreverted (seropositive animals at the beginning of the study which reverted to seronegative during the study), and animals with an intermittent presence of antibodies (alternating seropositive and seronegative status due to fluctuating antibody titers regardless of their serological status at the beginning of the study).

Morbidity frequency measures included prevalence (point and period), incidence rate, and cumulative incidence for MV seropositivity and were calculated as follows:

$$\mathrm{Point}\;{\mathrm{prevalence}}_{n}=\frac{seropositive\;ewes\;within\;n\;sampling\;occasion}{total\;ewes\;population\;within\;n\;sampling\;occasion}$$

$$\mathrm{Period}\;\mathrm{prevalence}=\frac{new\;and\;pre-existing\;cases\;of\;seropositive\;ewes\;during\;the\;study}{total\;ewes\;population\;during\;the\;study}$$

$$\mathrm{Incidence}\;\mathrm{rate}=\frac{new\;cases\;of\;seropositive\;ewes\;during\;the\;study}{sum\;of\;healthy\;sheep-semesters\;during\;the\;study}$$

$$\mathrm{Cumulative}\;\mathrm{incidence}=\frac{new\;cases\;of\;seropositive\;ewes\;during\;the\;study}{animals\;at\;risk\;at\;the\;beginning\;of\;the\;study\;(seronegative\;animals)}$$

For the calculation of the above-mentioned measures, the following assumptions were followed: (i) case was defined as the animal with seropositive status, (ii) a sheep was considered as new case once it was found seropositive, (iii) animals at risk referred as the seronegative animals at the beginning of the study, and (iv) for the calculation of the incidence rate, sheep-semester was defined as the unit of the time–person component. Each seronegative sheep contributed to the study of healthy sheep-semesters until found seropositive; once found seropositive, it did not contribute to any healthy sheep-semesters in the study even if seroreversion occurred.

Prevalence rates and 95% confidence intervals were calculated with R package *epi.prev* using Blaker method and adjusting for the sensitivity and specificity values of the ELISA test. The respective incidence values were calculated with R package *epi.conf* using Byar method for incidence rates and Wilson method for cumulative incidence rate. Descriptive statistics (frequencies) for various serological patterns, seroconversion, and seroreversion incidents were calculated in SPPS v.26.

## 3. Results

### 3.1. Age and Serological Status

**Figure 2:**
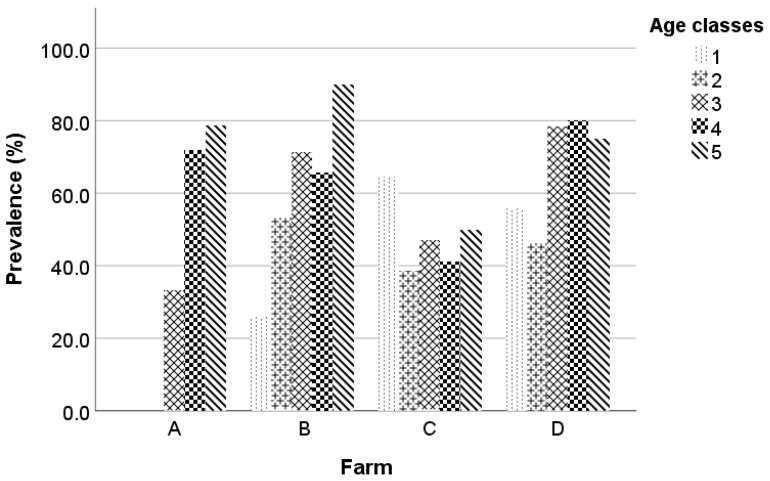
Maedi-visna seroprevalence at the beginning of the study per age class and farm; the five age classes are: 1 (x ≤ 1), 2 (1 < x ≤ 2), 3 (2 < x ≤ 3), 4 (3 < x ≤ 4), and 5 (x > 4).

The age of the studied animals at the beginning of the study ranged from 6 months to 7 years, with a mean value equal to 2.4 ± 1.46 years. The mean ages of Chios and Lacaune ewes were 2.7 ± 1.55 and 1.8 ± 1.16 years, respectively. The prevalence of MV at the beginning of the study was 49.1%, 47.6%, 61.3%, 63.5%, and 82.5% in the age categories 1 (x ≤ 1), 2 (1 < x ≤ 2), 3 (2 < x ≤ 3), 4 (3 < x ≤ 4), and 5 (x > 4), respectively. The prevalence of MV at the beginning of the study per age class and farm is in Figure 2.

**Figure 3:**
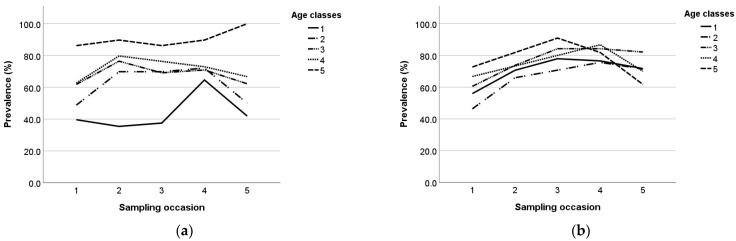
Maedi-visna seroprevalence during the study per age class in Chios (**a**) and Lacaune (**b**) breeds; the five age classes are: 1 (x ≤ 1), 2 (1 < x ≤ 2), 3 (2 < x ≤ 3), 4 (3 < x ≤ 4), and 5 (x > 4).

Seroprevalence during the study per age class and breed is presented in Figure 3a,b. During the study, the prevalence in age class 1 and 5 increased until the third sampling occasion and then decreased in Lacaune ewes, whereas in Chios ewes it fluctuated during the study. In age class 2, the prevalence followed a similar trend in both breeds; it increased until the fourth sampling and then decreased. Also, in age classes 3 and 4, the prevalence increased until the second sampling and then decreased in Chios ewes, whereas in Lacaune ewes, it increased until the fourth sampling and then decreased.

**Figure 4:**
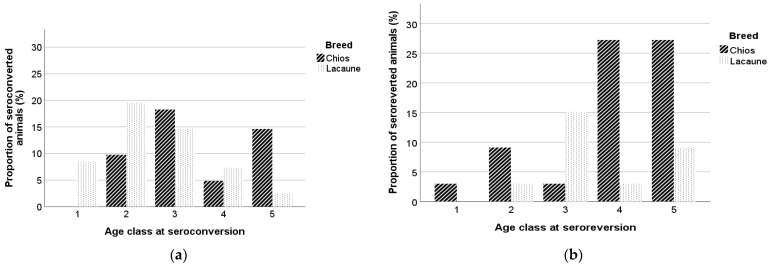
Age class of Chios and Lacaune ewes at seroconversion (**a**) and seroreversion (**b**) incident. Age is presented in year classes, 1 (x ≤ 1), 2 (1 < x ≤ 2), 3 (2 < x ≤ 3), 4 (3 < x ≤ 4), and 5 (x > 4).

At the beginning of the study, the mean ages of seropositive and seronegative animals were 2.6 ± 1.57 and 2.0 ± 1.24 years (3.1 ± 1.62 and 2.3 ± 1.33 for Chios and 2.0 ± 1.25 and 1.7 ± 1.01 for Lacaune ewes, respectively), and for constantly seronegative and constantly seropositive animals the mean ages were 1.9 ± 1.18 and 2.7 ± 1.62 years, respectively. The mean ages of animals which seroconverted were 2.0 ± 1.28 years at the beginning of the study and 2.9 ± 1.38 years at the seroconversion incident, while the mean ages for the seroreverted animals were 2.2 ± 1.27 years at the beginning of the study and 3.7 ± 1.47 years at the seroreversion incident. Sheep with an intermittent presence of antibodies had a mean age of 2.1 ± 1.17 years when introduced in the study. Among seroconverted animals, in 8.5%, 29.3%, 32.9%, 12.2%, and 17.1%, seroconversion occurred at the first, second, third, fourth, and greater than fourth year of age, while 70% of the seroreverted animals were more than three years old at the seroreversion incident. In Figure 4a,b, the mean ages of seroconversion and seroreversion in Chios and Lacaune breeds are presented.

### 3.2. Serological Patterns

**Figure 5:**
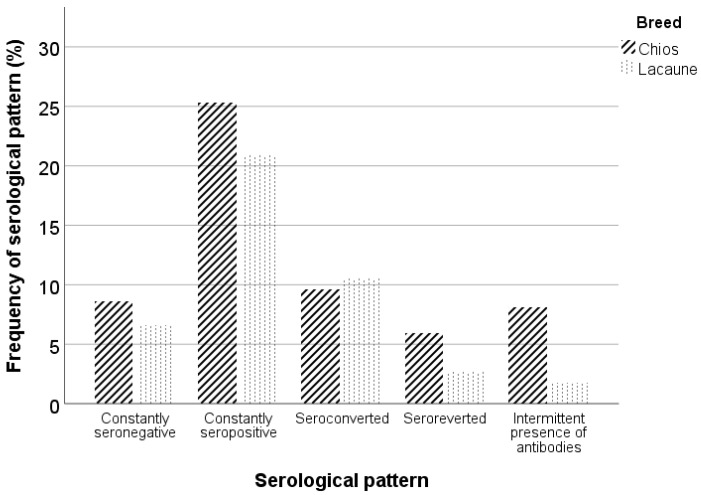
Serological patterns in Chios and Lacaune breeds.

All serological patterns were observed in each farm except for the constantly seronegative Chios sheep in farm A. Similar percentages of constantly seronegative and seropositive animals were observed in both breeds. However, the Lacaune breed demonstrated a higher percentage of seroconverted animals, and the Chios breed demonstrated higher percentages of seroreverted animals and animals with an intermittent presence of antibodies (Figure 5). In Table 2, frequencies of serological patterns per breed and farm are summarized.

**Figure 6:**
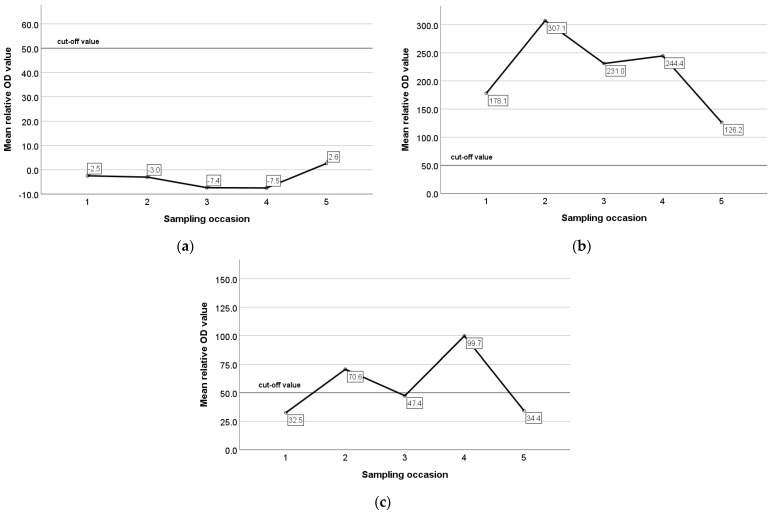
Mean relative optical density (OD) values of ELISA in constantly seronegative ewes (**a**), constantly seropositive ewes (**b**), and animals with an intermittent presence of antibodies (**c**) during the study.

**Figure 7:**
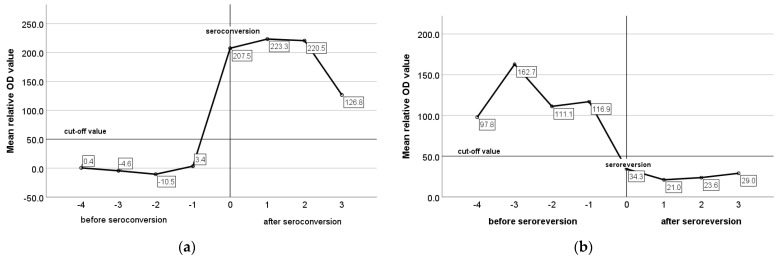
Mean relative OD values of ELISA in seroconverted (**a**) and seroreverted (**b**) ewes before and after the seroconversion/seroreversion.

The mean relative optical density (OD) values of ELISA for constantly seronegative, constantly seropositive, and animals with an intermittent presence of antibodies were −4.0, 224.1, and 58.2, respectively. In constantly seronegative animals, the mean relative OD values remained very low and did not present any remarkable variation during the study (Figure 6a), whereas in constantly seropositive animals and in animals with an intermittent presence of antibodies, the mean relative OD values were increased pre-lambing and decreased pre-mating (Figure 6b,c). In seroconverted and seroreverted ewes, and in ewes with an intermittent presence of antibodies, the mean relative OD values were 205.5, 126.6, and 110.6 for the seropositive status and −0.71, 29.4, 19.0. for the seronegative status, respectively. In Figure 7a,b, the mean relative OD values of seroconverted and seroreverted animals are presented before and after seroconversion/seroreversion.

### 3.3. Morbidity Frequency Measures

**Figure 8:**
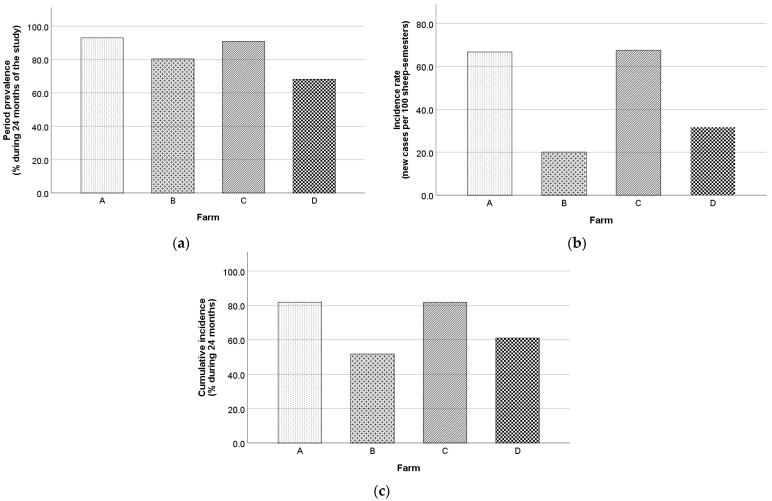
Maedi-visna period prevalence (**a**), incidence rate (**b**), and cumulative incidence (**c**) for each farm during the study.

A total of 345/407 animals (84.8%) were found seropositive at least once during the study, while 62/407 animals (15.2%) were constantly seronegative. During the study, the overall period prevalence, incidence rate, and cumulative incidence were 84.8% (95% CI, 80.9–88.0%), 33.6 new cases per 100 sheep-semesters (95% CI, 27.8–40.3%), and 64.2% (95% CI, 56.8–70.9%). The respective values for Chios and Lacaune ewes for the first year and the whole duration of the study are presented in Table 3. Also, farm B recorded the lowest values in all morbidity frequency measures, in contrast to farm A and C (Figure 8).

**Figure 9:**
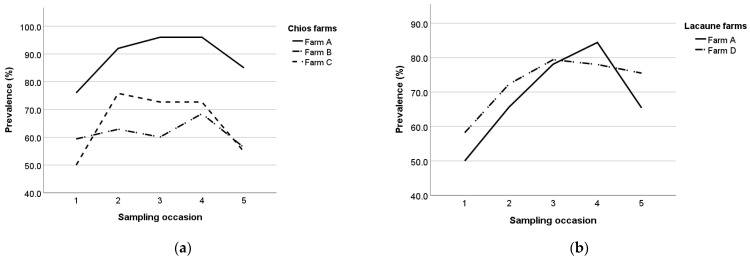
Maedi-visna seroprevalence in Chios (**a**) and Lacaune (**b**) farms during the study.

The point prevalence for each sampling occasion per breed and farm are presented in Table 4 and ranged from 48.5% (farm C, first sampling occasion) to 96.0% (farm A, fourth sampling occasion in Chios ewes). Farms B and C exhibited similar seropositivity evolution patterns with a consecutive increase and decrease between the sampling occasions, whereas in farms A and D, seropositivity was gradually increasing before a final decrease in the last sampling occasion (Figure 9).

## 4. Discussion

To our knowledge, this is the first prospective seroepidemiological study of MV in intensively reared dairy sheep flocks of two of the most productive dairy sheep breeds in Greece, namely Chios and Lacaune. Also, it is the first time that seroreversion incidents and cases of an intermittent presence of antibodies are systematically recorded in MV naturally infected ewes, supporting the notion of introducing serological patterns for the classification of animals rather than their typical classification into seropositive and seronegative.

Our findings confirm the hypothesis of increased MV prevalence in intensively reared dairy sheep flocks in Greece. This is in accordance with the results from other Mediterranean countries with a developed dairy sheep sector (e.g., Spain, Italy, etc.) [15,16,22]. Before this study, limited epidemiological evidence of MV prevalence in our country was available through one cross-sectional study, which included 143 sheep from six infected flocks and was aimed at the serological and molecular detection of MV infection [23], and one prospective study (>25 years ago) in which 378 Chios ewes originating from the experimental flock of the Animal Research Institute (ELGO-Demeter) were followed for one lactation to assess the effect of maedi-visna seropositivity on the milk yield [7]. The seroprevalence rates in the forementioned studies were 65.0% and 47.0%, respectively, and in any case similar to our results (57.7–75.4%). Increased prevalence rates of MV in intensive farms of high-yielding dairy breeds in our country indicate that the production of MV-free animals will be a critical challenge for the local breeding stocks market in the future, given that these farms are key players in this market.

The point seroprevalence found in our study in each sampling occasion for the total of studied animals ranged from 57.5% to 75.4% and is higher compared to the seroprevalence documented in recent cross-sectional epidemiological studies utilizing ELISA testing in other countries. In particular, low seroprevalence rates were found in Poland (5.4–14.9% in a population of 6470 ewes from 98 flocks) [18], in Croatia (10.0% in 460 sheep from 17 farms) [11], in Belgium (9.0% in 555 sheep from 87 farms) [9], and in Turkey (15.3% in Istanbul and 5.7% in Afyonkarahisar in 542 sheep from 4 flocks and 248 sheep from 22 flocks, respectively) [24,25]. Moreover, in Japan, an epidemiological survey in 267 adult sheep from 14 sheep flocks using both AGID and ELISA tests revealed only three seropositive animals, confirming the limited spread of MVV in the country [26]. Medium prevalence rates were observed in Germany (28.8% in 2229 sheep from 41 farms) [14], in Canada (32.0% in 1954 sheep from 29 farms) [5], and in Iran (34.5% in 220 sheep from 30 flocks) [10], while in Spain and China seroprevalence rates ranged from medium to high among the different studied regions (24.8–54.4% in three studies in Spain including 15,155 sheep from 78 flocks, 274,048 sheep from 554 flocks, and 5120 sheep from 239 flocks as well as 4.6–50.0% in China in 672 sheep from 24 flocks) [15,16,27,28]. Nevertheless, direct comparisons between these seroprevalence values and the respective found in our study are rather arbitrary, as the vast majority of these studies are cross-sectional and include sheep population of many breeds, reared in various farms of unknown seropositivity status and under different production systems (from extensive to intensive). On the other hand, in our study the farms were selected using specific criteria; one criterion was the seropositive status at the farm level, which was necessary for the assessment of the morbidity frequency measures (prevalence, incidence rate, and cumulative incidence) during the two-year period and at given stages of the production cycle. Another criterion was zero-grazing, intensive management, where animals are more exposed to MVV infections due to closer contact followed by extensive horizontal transmission of the MVV [16,29,30]. Moreover, the increased seroprevalence found herein could partially be attributed to the type of ELISA used for the serological testing, which has the whole virus as an antigen, therefore increasing the detection spectrum of the specific antibodies and, subsequently, the sensitivity and the overall performance of the assay.

Longitudinal studies investigating the morbidity frequency measures of MV are limited; thereby, the benchmarking of our results with similar studies to comparatively assess and update the current situation in various occasions is not feasible. The prevalence and incidence rates observed in our study coincide with those found in a prospective study on intensively reared Assaf sheep by Leginagoikoa et al. [31]. On the contrary, the overall cumulative incidence rate found herein was higher (64.2%) compared to studies on semi-intensively reared Latxa breed flocks (19.6% and 27.0%) [32,33]. However, in the latter studies, the cumulative incidence rate was calculated for young animals from the post-weaning, whereas in our study, all the age classes of animals and mainly the older ones were represented. In the present study, the period prevalence and incidence rate during the first year and the whole duration of the study were increasing, indicating the significant role of late seroconversion and/or of the horizontal transmission for the spread of the infection within the herd. Period prevalence increased by ca. 18.0% in the first year of the study and increased further by 10% in the second year, suggesting that MV prevalence gradually reaches a plateau, with constant seronegative animals (ca. 15%) being found either due to unsuccessful seroconversion in infected animals or due to a potential underlying genetic resistance against MV infection [34,35].

To the best of our knowledge, this is the first time different serological patterns, as determined by the seroconversion and seroreversion events, are defined and described in MV naturally infected sheep under field conditions in a large-scale epidemiological study. In our study, 9.8% (40/407) of animals presented an intermittent presence of antibodies and 8.6% (35/407) seroreverted. The presence of these serological patterns is not likely to result from the imperfect diagnostic performance of the ELISA test; in fact, all serological analyses were performed by the same trained veterinarian in a single laboratory, using the same equipment and protocols, shortly after the blood samplings to avoid inconsistencies and minimize the possibilities of diagnostic errors and the misclassification of animals. The proportion of seroreverted animals and animals with an intermittent presence were rather high to be hypothesized and could be due to false negative results. This is also supported by the fact that more than 50% of the animals in these categories demonstrated a specific serological pattern during the study, which was not determined by a single seroconversion or seroreversion event; seroreverted animals presented negative results more than once after consecutive positive results, and animals with an intermittent presence of antibodies had an alternating serological status between sampling occasions. However, the possibility of circulating more than one strain in each herd cannot be excluded, and thus the produced neutralizing antibodies could perform a different affinity with the antigens of the applied ELISA test.

Seroreversion reactions were previously reported in studies with a limited number of sheep and goats, with lambs and goat kids where the transient presence of maternal antibodies constitutes a confounding factor, with experimentally infected goats where their immune response is lower compared to naturally infected ones [36], or with cases of advanced MV disease [37] without clearly suggesting the mechanism behind this serological reaction. Seroreversion was described in HIV infected adults and children treated with antiretroviral therapy after acute infection [38,39,40,41,42,43] and in end-stage HIV patients [44]; in the first case, it results from long-lasting viral suppression under undetectable limits, whereas in the second case the most possible explanation is the loss of antibodies against capsid proteins. In our study, animals were infected and maintained the seropositive status for a long period before the seroreversion incident. Moreover, the mean relative OD values of seroreverted animals and animals with an intermittent presence of antibodies in a seronegative status remained relatively high and closer to the threshold of the ELISA test (28.91 and 18.50, respectively), compared to the constantly seronegative animals (−1.45). This finding could be used as an indicator of the occurrence of seroreversion rather than a seronegative status, a hypothesis, which needs to be evidenced in the future.

The mean relative OD values of animals with an intermittent presence of antibodies were below the threshold pre-mating and were above it pre-lambing, suggesting an underlying mechanism in humoral immune response that is probably associated with late pregnancy and physiological changes. Seroreversion in our study could be associated with a suppression of neutralizing antibodies, related to an adverse overall health status, or with undefined peculiarities of humoral immune response in infected sheep. The cases of seroreverted ewes and of ewes with an intermittent presence of antibodies herein are currently under further investigation with molecular testing for the definite causative diagnosis and the elucidation of the mechanism which leads to this decline in antibody titers (unpublished data).

The occurrence of various serological patterns of infected animals determined with seroconversion and seroreversion events implies that it is likely that seronegative animals can be misdiagnosed as non-infected in cross-sectional studies. In that case, it is impossible to efficiently apply MV control programs, as seroreverted animals are grouped with the uninfected animals, continuously spreading MVV. This could at least partially explain the failure of current control programs to eradicate MV in specific regions (which was mainly attributed to the low sensitivity of serological assays), highlighting the significance of consecutive screening controls in infected flocks. A total of 15.2% (62/407) of the studied ewes were constantly seronegative. The identification of animals assigned to the specific serological pattern and the investigation of its association with genetic resistance (e.g., *TMEM154*, *CCR5*, and *DPPA2* genes) could be of value in genetic selection programs designed for the elimination of the disease. However, in that case, molecular techniques for the virus detection need to be jointly utilized, as the possibility of late seroconversion, seroreversion, or false negative results cannot be excluded [36].

Increased age was investigated as risk factor in several studies, and its significance was sufficiently documented [5,10,16,33]. The association between age and seroconversion could be attributed either to the late immune response in infected animals (months to years after the infection) [45,46] or to the increased risk of infection in older animals due to a longer exposure to the virus compared to younger animals. This is in consistency with our results, where seroprevalence was increased in older age classes at the beginning of the study and continued to increase in animals less than three years old, whereas it was stable or slightly declined in older animals. The decrease in seroprevalence in the last sampling occasion in all age classes could be attributed to the removal of seropositive animals between the fourth and the fifth samplings. Τhe stabilization of seroprevalence rates in animals older than three years old indicates that (i) in most cases, seroconversion occurs before three years old whereas seroreversion occurs in animals older than three years old in farms with high MV prevalence because animals are exposed to a high viral load at birth; (ii) animals resistant to the infection or infected animals which do not seroconvert are likely.

Although this prospective study of MV seroprevalence allows the extraction of important conclusions compared to the currently available cross-sectional studies, the current study design makes the generalization of the results infeasible. For example, we studied only purebred Chios and Lacaune ewes, from intensive and proven MV-infected farms. Further, large-scale studies undertaken in different breeds and farming systems, and in farms with various seroprevalence rates, are necessary to confirm the universal applicability of our findings. Based on the acquired results, the next steps should be (i) the molecular testing of the studied animals and the comparison with a respective serological test to conclude the seronegative, the seroreverted, or the false-negative status, and (ii) the comparison of production and health traits between seronegative and seropositive animals and among animals belonging to different serological patterns.

## 5. Conclusions

Our results suggest high seroprevalence rates of maedi-visna in intensive dairy sheep farms of purebred Lacaune and Chios sheep in Greece, although large-scale epidemiological studies including more breeds reared under different production systems are warranted for the reliable estimation of virus spread in the country. High incidence rates among adult ewes in our study indicate the necessity for establishing stricter preventive and eradication measures against horizontal transmission in infected farms. Contrary to what is known, a non-negligible proportion of infected animals manifest different serological patterns beyond the known constant seropositive or seronegative ones, such as the seroreversion or the intermittent presence of antibodies, hence leading to the misdiagnosis of infections. These serological reactions need to be further investigated and elucidated regarding their association with a clinical manifestation of the MV disease or viral eradication. Our results confirm the necessity for continuous screening controls until the eradication of MV in infected flocks, as the cross-sectional testing is likely to fail in detecting the total of infected animals. Also, the presence of constantly seronegative animals in farms with a high MV prevalence strongly indicates a potential for genetic resistance which should be investigated and exploited in control programs based on genetic selection.

## Figures and Tables

**Table 1:** Farm characteristics and herd management practices in the studied farms.

	Farm A	Farm B	Farm C	Farm D
Location	Larissa	Preveza	Korinthos	Aetolia-Acarnania
Breed	Chios/Lacaune	Chios	Chios	Lacaune
Years of operation	7	7	4	9
Animals per employee (n)	118	100	224	115
Total animal number (n)	241	208	925	230
Milking ewes (n)	156	160	615	148
Replacement rate (%)	30	25	40	25
Shed area/ewe (m^2^)	1.42	2.70	1.53	1.50
Shed volume/ewe (m^3^)	7.10	13.50	12.24	5.20
Type of ventilation	Natural	Natural	Natural and mechanical	Natural and mechanical
Ventilation conditions	Poor	Good	Very good	Medium
Type of bedding	Straw	Straw	Straw	Straw
Frequency of manure removal (times/year)	1	3	6	1
Feeder space/sheep (cm)	20.7	31.3	35.9	34.4
Exercise paddock	No	Yes	No	Yes
Method of mating	Natural/groups	Natural/single sire groups	Natural/groups	Natural/single sire groups
Ewes:rams ratio	20	20	24	24
Milk yield/ewe/lactation 210 days (kg)	300	400	250	450
Prolificacy (lambs/ewe)	1.4	2	1.9	1.6
Method of lamb rearing	Natural	Artificial	Artificial	Artificial
Weaning age (days)	40	45	50	35
Method/frequency of milking (times/day)	Mechanical/2	Mechanical/3-2	Mechanical/3-2	Mechanical/2
Vaccinations—treatments:				
Enterotoxemia	✓	✓	✓	✓
Pasteurellosis	−	−	✓	−
Contagious agalactia	✓	✓	✓	✓
Enzootic abortion	✓	✓	✓	✓
Anthelmintic treatment	✓	✓	✓	✓
Dry-off treatment	−	✓	✓	✓
Health issues:				
Mastitis	<10%	5%	10%	<10%
Abortions	<5%	<5%	5%	<5%
Lameness	<5%	<5%	<5%	5%
Pregnancy toxemia	<5%	<5%	<5%	<5%
Confirmed maedi-visna clinical cases	No	No	No	No

**Table 2:** Serological patterns in each farm and overall.

Serological Pattern	Chios			Lacaune			
	Farm A	Farm B	Farm C	Farm A	Farm D	All Farms	
Constantly seronegative	0.0% (0/25)	19.6% (28/143)	10.6% (7/66)	12.5% (4/32)	16.3% (23/141)	15.2% (62/407)	
Constantly seropositive	64.0% (16/25)	42.7% (61/143)	39.4% (26/66)	37.5% (12/32)	51.8% (73/141)	46.2% (188/407)	
Seroconverted	20.0% (5/25)	14.0% (20/143)	21.2% (14/66)	31.3% (10/32)	23.4% (33/141)	20.1% (82/407)	
Seroreverted	12.0% (3/25)	11.2% (16/143)	7.6% (5/66)	12.5% (4/32)	5.0% (7/141)	8.6% (35/407)	
Intermittent presence of antibodies	4.0% (1/25)	12.6% (18/143)	21.2% (14/66)	6.3% (2/32)	3.5% (5/141)	9.8% (40/407)	

**Table 3:** Period prevalence, incidence rate, and cumulative incidence (95% CI) for Chios and Lacaune ewes.

Morbidity Frequency Measure	Chios		Lacaune	
	12 months	24 months	12 months	24 months
Period prevalence (%)	73.1 (66.9–78.5)	84.6 (79.4–88.8)	81.5 (75.0–86.9)	84.4 (78.2–89.2)
Incidence rate (new cases per 100 sheep-semesters)	37.8 (27.9–50.1)	32.8 (25.4–41.7)	51.2 (37.4–68.5)	34.8 (26.0–45.7)
Cumulative incidence (%)	45.9 (36.4–55.8)	64.3 (54.4–73.1)	56.0 (44.7–66.7)	64.0 (52.7–73.9)

CI: confidence interval.

**Table 4:** Point seroprevalence for each breed and for the total of studied animals throughout the study.

Sampling Occasion	Prevalence		
	Chios Ewes	Lacaune Ewes	Overall
1st (pre-mating)	58.2% (136/234)	56.6% (98/173)	57.5% (234/407)
2nd (pre-lambing)	69.2% (162/234)	71.1% (123/173)	70.0% (285/407)
3rd (pre-mating)	66.7% (156/234)	78.6% (136/173)	71.7% (292/407)
4th (pre-lambing)	72.6% (170/234)	79.2% (137/173)	75.4% (307/407)
5th (pre-mating)	59.7% (92/154)	73.4% (94/128)	66.0% (186/282)

## Data Availability

The data presented in this study are available within the article. Raw data supporting this study are available from the corresponding author upon reasonable request.
